# Development of a risk prediction model for central venous catheter insertion-related thrombosis in critically ill pediatric patients

**DOI:** 10.3389/fped.2026.1666896

**Published:** 2026-03-24

**Authors:** Xiaolan Zhou, Yunrong Li, Shoushan Chen, Yingbo Zou, Huan Wang, Li Lei

**Affiliations:** Pediatric Intensive Care Unit, The First People’s Hospital of Zunyi (The Third Affiliated Hospital of Zunyi Medical University), Zunyi, Guizhou, China

**Keywords:** central venous catheters, critically ill child, nomograms, risk factors, venous thrombosis

## Abstract

**Introduction:**

Central venous catheter-related thrombosis (CVC-RT) is a serious complication associated with CVC insertion that significantly adversely affects the prognosis of critically ill children. This study aimed to identify risk factors for CVC-RT following CVC placement in critically ill children and to develop a corresponding risk prediction model.

**Methods:**

A total of 188 critically ill children with CVCs were enrolled and categorized into thrombosis and non-thrombosis groups. Clinical data were collected to analyze risk factors for CVC-RT, and a nomogram prediction model was developed and validated for its predictive performance.

**Results:**

Among the 188 children, 31 developed CVC-RT, yielding an incidence rate of 16.5%. Significant differences were observed between the two groups in terms of age, catheter type, parenteral nutrition status, D-dimer levels, and fibrinogen (FIB) levels. All of these factors, except catheter type, were identified as independent predictors of CVC-RT. The constructed nomogram prediction model demonstrated strong predictive performance and discriminative ability, with an area under the receiver operating characteristic curve of 0.952.

**Discussion:**

In summary, this study identified age, parenteral nutrition, D-dimer, and FIB levels as independent influencing factors for CVC-RT in critically ill children. The nomogram model incorporating these factors exhibited favorable predictive value.

## 1 Introduction

Central venous catheter (CVC) placement is an indispensable intervention in the pediatric intensive care unit (PICU), indicated for patients with hemodynamic instability or those requiring parenteral nutrition (1–3). CVCs are widely utilized in pediatric critical care settings, with up to 69% of hospitalizations in pediatric cardiac ICUs involving CVC placement— a rate as high as 86% among neonates (4). Despite its clinical importance, CVC insertion carries a risk of serious complications, among which central venous catheter-related thrombosis (CVC-RT) is particularly concerning due to its potential life-threatening implications. CVC-RT can cause catheter dysfunction, prolong hospital stays, and increase medical costs, highlighting the urgent need for effective risk prediction models to guide preventive strategies (5).

The burden of CVC-RT in the pediatric critical care setting is substantial (6). A study analyzing 50 patients admitted to the PICU reported that the proportion of patients who developed CVC-RT was as high as 34% (7). Large multicenter studies have further confirmed this burden: the CATCH trial, involving 1,485 children across 14 PICUs, reported an overall CVC-RT incidence of 24%, with 12.1% classified as clinically relevant (8). Compared with adults, the pathophysiology of CVC-RT in children is more complex, influenced by factors such as smaller vessel diameter, hemodynamic instability, and disease-specific risks, which complicate clinical management (9).

Currently, most CVC-RT risk prediction models—such as the Khorana score and the CATS model—are derived from adult populations and rely on coagulation markers and catheter-related parameters (10, 11). However, these models may not be directly applicable to pediatric patients due to distinct physiological and clinical characteristics. Several researchers have developed pediatric-specific risk prediction models for CVC-RT (12, 13). However, existing pediatric VTE risk screening guidelines have low sensitivity (approximately 61%), failing to identify nearly 40% of CADVT events, while available risk assessment models lack multicenter validation and broad applicability (14, 15). Although recent single-center retrospective studies have sought to identify risk factors for CVC-RT in children, a widely applicable predictive tool remains unavailable, leaving clinical prevention strategies inadequately supported (16, 17). Therefore, preventing and managing CVC-RT continues to pose a major challenge in pediatric practice, underscoring the need for a reliable risk prediction model to facilitate early identification of high-risk patients and support individualized prophylaxis.

In this study, we adopted a retrospective case-control design to integrate demographic and catheterization-related parameters in critically ill children. We aimed to identify independent risk factors for CVC-RT and develop a nomogram prediction model to provide a quantitative tool for early risk assessment and prevention of CVC-RT in this vulnerable population.

## 2 Methods

### 2.1 Study population and catheterization procedures

This was a retrospective cohort study conducted at Zunyi First People's Hospital. A total of 188 critically ill children who underwent CVC placement between August 2023 and December 2024 were enrolled. All children were admitted to the PICU of our hospital. CVC insertions were performed by attending physicians or senior physicians in the PICU, all of whom possessed over five years of clinical experience and had completed specialized training in central venous catheterization. The right internal jugular vein was the preferred site, followed by the subclavian vein; the femoral vein was used only when the other sites were unsuitable. The selection of puncture site was based on ultrasound assessment of the vasculature, clinical requirements, and other relevant factors.

All CVC insertions were performed under ultrasound guidance at the bedside in the PICU, except for children with poor vascular conditions (e.g., small vessel caliber, anatomical variations), in whom the procedure was conducted in the interventional radiology department. No insertions required the operating room. Before catheterization, the diameter of the target vein was measured by ultrasound, and the principle of “catheter diameter ≤ 2/3 of the venous diameter” was strictly followed to avoid excessive compression of the vessel wall. The catheters used in this study were supplied by Able (Guangzhou, China). The catheter types included 1.9 F (dual-lumen), 2.6 F (dual-lumen), and 6.6 F (single-lumen).

Patients were divided into a non-thrombosis group (*n* = 157) and a thrombosis group (*n* = 31) based on the occurrence of CVC-RT. All patients met the following inclusion criteria: (1) age ≤ 6 years; (2) successful insertion of a CVC based on clinical indications; (3) availability of complete medical records. In the retrospective data collection, we excluded the following cases: (1) presence of severe bleeding disorders; (2) catheters that require immediate removal or repositioning after insertion due to technical complications; (3) preexisting prethrombotic state, defined as either congenital coagulation abnormalities (e.g., antithrombin deficiency, protein C/S deficiency, confirmed by genetic or laboratory testing) or acquired hypercoagulable states (e.g., Kawasaki disease, nephrotic syndrome, confirmed by clinical diagnosis); (4) cases with peripherally inserted central catheters (PICC).

Upon diagnosis of CVC-RT, most catheters were removed or replaced based on clinical assessment. In this study, the catheter dwell time in the thrombosis group was counted up to the date of thrombosis diagnosis.

### 2.2 Data collection

Data were retrospectively extracted from the hospital electronic medical record system, including: (1) basic patient information: age, sex, height, and weight, to establish baseline characteristics; (2) surgical information: type of surgery, duration of operation, and surgical approach, to evaluate the impact of surgical factors on CVC-RT risk; (3) catheter-related parameters: insertion site, catheter type, indwelling time, and insertion technique, to assess the influence of catheterization factors on CVC-RT risk; (4) preprocedural laboratory values: levels of D-dimer and fibrinogen (FIB) measured prior to CVC insertion; (5) nutritional support status: whether the patient received total parenteral nutrition during the catheter indwelling period. In accordance with the standard protocol of our PICU, no routine prophylactic anticoagulation was administered to any patient in this study.

### 2.3 Diagnostic criteria for CVC-RT

CVC-RT was diagnosed if at least one clinical symptom (criteria 1 or 2) plus Doppler ultrasound evidence (criterion 3) were fulfilled:

1. Swelling, pain, or increased local skin temperature in the limb on the catheterized side;
2. Presence of localized erythema, tenderness, or dilation of collateral veins;
3. Doppler ultrasound evidence of venous lumen enlargement with hypoechoic or hyperechoic thrombus, accompanied by narrowing or absence of color flow signals.

### 2.4 Statistical analysis

Statistical analyses were performed using SPSS 22.0 and R software. Continuous variables were tested for normality using the Kolmogorov–Smirnov test, and homogeneity of variance was assessed with Levene's test. Normally distributed data were expressed as mean ± standard deviation (x¯ ± s) and compared using the t-test. Categorical data were expressed as frequencies and percentages [*n* (%)] and analyzed with the chi-square test. For time-to-event analysis of CVC-RT-free survival, Kaplan–Meier curves were plotted to estimate survival probabilities, and differences between stratified groups were compared using the log-rank test. Variables that showed statistically significant differences in univariate analyses between the thrombosis and non-thrombosis groups were included in a multivariate logistic regression analysis. A nomogram was developed using the rms package in R to identify factors associated with CVC-RT. The predictive performance of the model was evaluated using receiver operating characteristic (ROC) curve analysis, and clinical utility was assessed via decision curve analysis. An area under the curve (AUC) greater than 0.9 was considered to indicate high predictive accuracy. A two-tailed *P*-value < 0.05 was considered statistically significant.

## 3 Results

### 3.1 Univariate analysis of CVC-RT occurrence in critically ill children

A total of 188 critically ill children with centrally inserted central venous catheters (CVC) were included in this analysis. Among the included patients, 31 developed CVC-RT, corresponding to an incidence rate of 16.5%. All of these patients had their catheters removed or replaced after the diagnosis of CVC-RT. The median time to thrombosis occurrence was 14.1 days. The total number of CVC-days was 2,632, resulting in an incidence density of 11.78 per 1,000 catheter-days.

As summarized in Tables 1, 2, age, catheter type, use of parenteral nutrition, D-dimer levels, and FIB levels showed statistically significant differences between the thrombosis and non-thrombosis groups, suggesting their potential association with CVC-RT development. In contrast, no significant differences were observed between the two groups in terms of catheter side, catheterization duration, number of lumens, catheter-related infection, insertion site, or other baseline characteristics. Notably, 23 patients received 6.6 Fr large-bore catheters, which were selected for unavoidable clinical indications including acute kidney injury requiring continuous renal replacement therapy (CRRT) or severe hypovolemic shock needing rapid fluid resuscitation. Subgroup analysis of these patients (Supplementary Table S1) showed a CVC-RT incidence of 30.4% (7/23), with the thrombosis subgroup having a significantly younger age (0.63 ± 0.23 years vs. 4.99 ± 0.55 years, *P* < 0.0001). No significant differences in gender, catheterized side, or other parameters were observed between the thrombosis and non-thrombosis subgroups of 6.6Fr catheter users.

**Table 1:** Comparison of baseline characteristics in critically ill children [*n* (%)] (x¯ ± s).

Project	Non-thrombosis group (*n* = 157)	Thrombosis group (*n* = 31)	Z/*χ*2/t	*P* value
Age (years)	4.96 ± 0.52	0.82 ± 0.29	42.92	<0.0001
Gender			0.1317	0.895
Male	78 (49.7%)	15 (48.4%)		
Female	79 (50.3%)	16 (51.6%)		
Disease type			3.880	0.5668
Nervous system	58 (36.9%)	11 (35.5%)		
Respiratory system	34 (21.7%)	6 (19.4%)		
Sepsis	23 (14.6%)	7 (22.6%)		
Cardiovascular system	18 (11.5%)	2 (6.5%)		
Tumor	4 (2.5%)	2 (6.5%)		
Others	20 (12.7%)	2 (6.5%)		

**Table 2:** Univariate analysis of CVC-RT occurrence in critically ill children [*n* (%)] (x ± s).

Project	Non-thrombosis group (*n* = 157)	Thrombosis group (*n* = 31)	Z/χ2/t	*P* value
Catheterized side			0.5303	0.5959
Left	48 (30.6%)	8 (25.8%)		
Right	109 (69.4%)	23 (74.2%)		
Catheter type			6.648	0.0360
1.9f	78 (49.7%)	18 (58.1%)		
2.6f	63 (40.1%)	6 (19.4%)		
6.6f	16 (10.2%)	7 (22.5%)		
Catheterization time (days)	14.25 ± 3.31	13.93 ± 2.92	0.5022	0.6161
Catheter lumen			1.924	0.0544
Single-lumen	16 (10.2%)	7 (22.6%)		
Dual-lumen	141 (89.8%)	24 (77.4%)		
Parenteral nutrition			2.114	0.0345
Yes	18 (11.5%)	8 (25.8%)		
No	139 (88.5%)	23 (74.2%)		
Catheter-related infection			0.8779	0.3800
Yes	5 (3.2%)	2 (6.5%)		
No	152 (96.8%)	29 (93.5%)		
Catheter insertion site			1.325	0.5155
Internal jugular vein	65 (41.4%)	16 (16.2%)		
Subclavian vein	55 (35.0%)	10 (6.4%)		
Femoral vein	37 (23.6%)	5 (3.2%)		
D-dimer(mg/L)	0.46 ± 0.12	0.59 ± 0.13	5.371	<0.0001
FIB(g/L)	3.97 ± 1.14	4.80 ± 1.22	3.670	0.0003

Catheterization time refers to the total dwell time of the catheter from insertion to removal.

**Figure 1:**
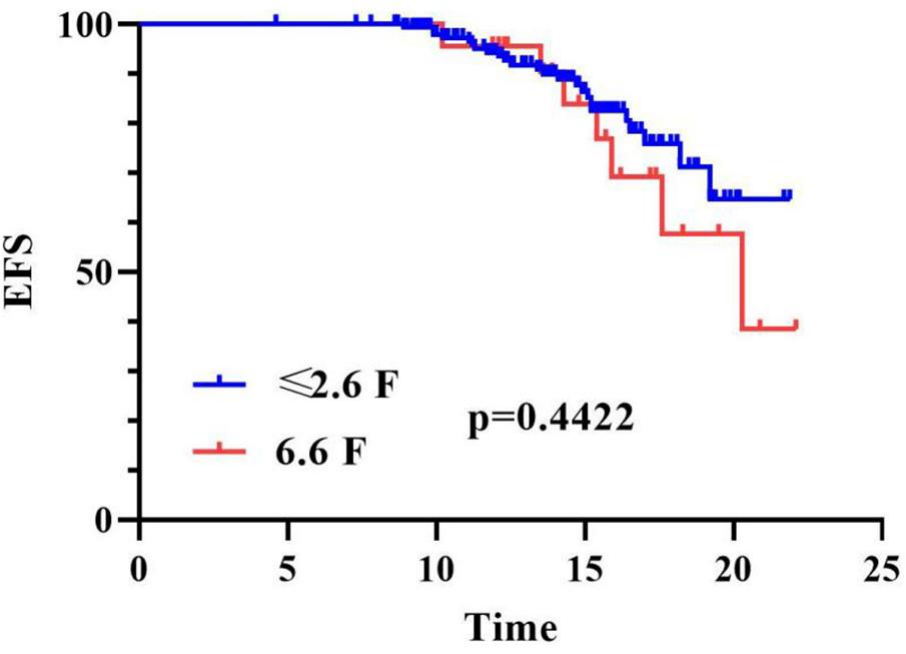
Kaplan–Meier curves of CVC-RT-free survival stratified by catheter diameter in critically ill children. EFS, event-free survival, the time from CVC insertion to the occurrence of CVC-RT or catheter removal in this study; *X*-axis, Time (days); *Y*-axis: CVC-RT-free survival rate (%).

**Figure 2:**
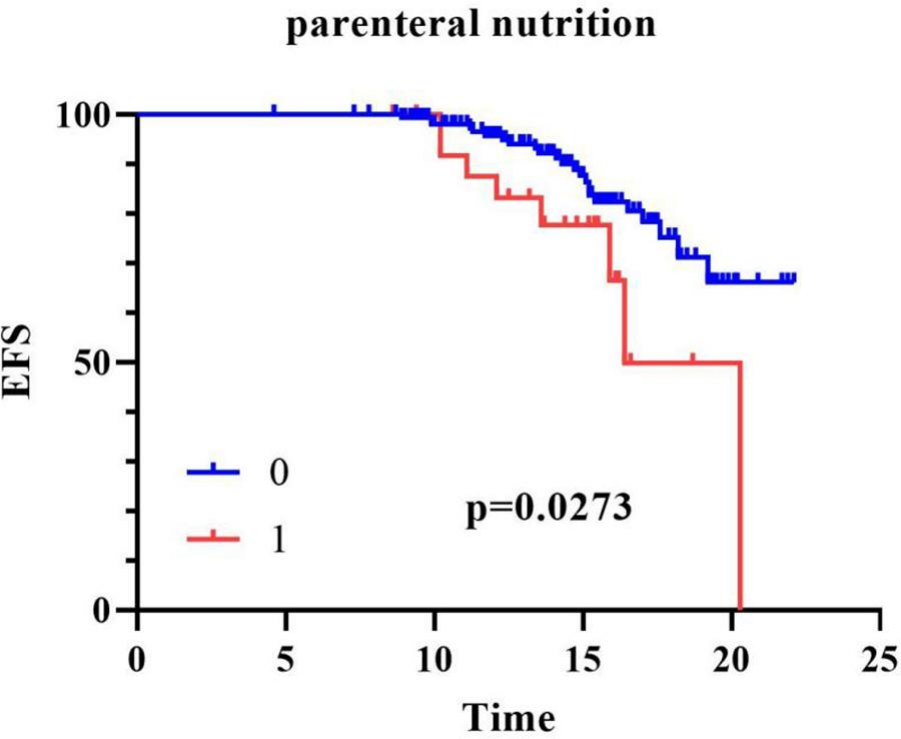
Kaplan–Meier curves of CVC-RT-free survival stratified by parenteral nutrition status in critically ill children. EFS, event-free survival, the time from CVC insertion to the occurrence of CVC-RT or catheter removal in this study; *X*-axis, Time (days); *Y*-axis: CVC-RT-free survival rate (%).

To evaluate the impact of catheter dwell time on the incidence of CVC-RT, we constructed thrombosis-free survival curves using the Kaplan–Meier method and compared them with the log-rank test. Stratification by catheter diameter (≤2.6 F vs. 6.6 F) showed that the 6.6 F group exhibited a steeper decline in thrombosis-free survival; however, the difference between the two groups was not statistically significant (Figure 1, log-rank *p* = 0.4422). In contrast, stratification by parenteral nutrition status revealed a significantly higher risk of thrombosis in patients receiving parenteral nutrition (Figure 2, log-rank *p* = 0.0273). These analyses confirm that parenteral nutrition significantly influences the cumulative incidence of CVC-RT over time.

### 3.2 Logistic regression analysis of risk factors for CVC-RT in critically ill children

Multivariable logistic regression was conducted to identify independent risk factors for CVC-RT in critically ill children. The dependent variable was the occurrence of CVC-RT (coded as 0 = absent, 1 = present). Independent variables that showed significant associations in univariate analyses were included in the model; their specific coding schemes are provided in Supplementary Table S2. As shown in Table 3, the analysis identified patient age, receipt of parenteral nutrition, D-dimer level, and FIB level as independent predictors of CVC-RT.

**Table 3:** Multivariate logistic regression analysis of CVC-RT occurrence in critically ill children.

Independent variable	*β*	SE	OR	Wald χ2	95%CI	*P*
Age	−1.543	0.355	0.214	18.919	0.107–0.428	0.0001
Catheter type	−0.464	0.387	0.629	1.438	0.295–1.342	0.230
Parenteral nutrition	2.344	0.859	10.424	7.441	1.934–56.175	0.006
D-dimer (mg/L)	11.804	3.148	1.34e + 5	14.064	280.1–6.40e + 7	0.0001
FIB (g/L)	0.545	0.262	1.724	4.330	1.032–2.880	0.037

Moreover, after excluding the cohort of patients with 6.6 Fr catheters, additional independent analyses were conducted. Univariate analysis revealed that age, D-dimer levels, and FIB levels remained significantly different between the thrombosis and non-thrombosis groups (all *P* < 0.05), while no statistical differences were found in catheter type, use of parenteral nutrition, catheter side, catheterization duration, or catheter-related infection (all *P* > 0.05, Supplementary Table S3). Further multivariate Logistic regression analysis identified age as the only independent risk factor for CVC-RT in critically ill children (*P* = 0.033). The assignment of independent variables is provided in Supplementary Table S4. In contrast, catheter type (*P* = 0.789), D-dimer levels (*P* = 0.551), and FIB levels (*P* = 0.717) did not show a statistically significant independent association with CVC-RT development after adjusting for potential confounders (Supplementary Table S5).

### 3.3 Construction and evaluation of a nomogram prediction model for CVC-RT in critically ill children

**Figure 3:**
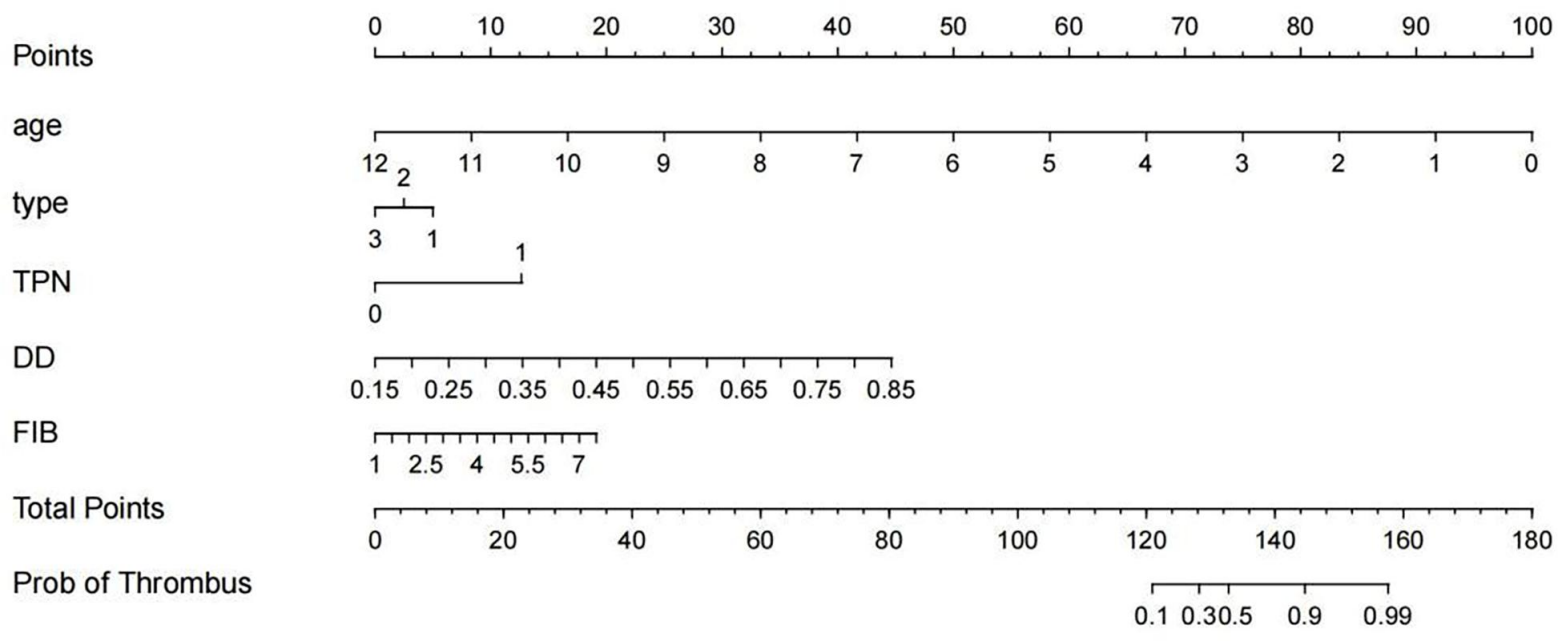
Nomogram prediction model for CVC-RT in critically ill children. TPN, total parenteral nutrition; DD, D-dimer; FIB, fibrinogen. Axis units: Age: years; DD: mg/L; FIB: g/L; Probability of Thrombus: 0–1.

A nomogram model was developed to predict the risk of CVC-RT in critically ill children based on four independent predictors: age, parenteral nutrition, D-dimer, and FIB levels. In the nomogram, the total points derived from these variables correspond to the predicted probability of CVC-RT occurrence. For instance, a total score of 120 points corresponded to a CVC-RT probability of 10%, while a score around 160 points indicated a probability of approximately 99% (Figure 3).

**Figure 4:**
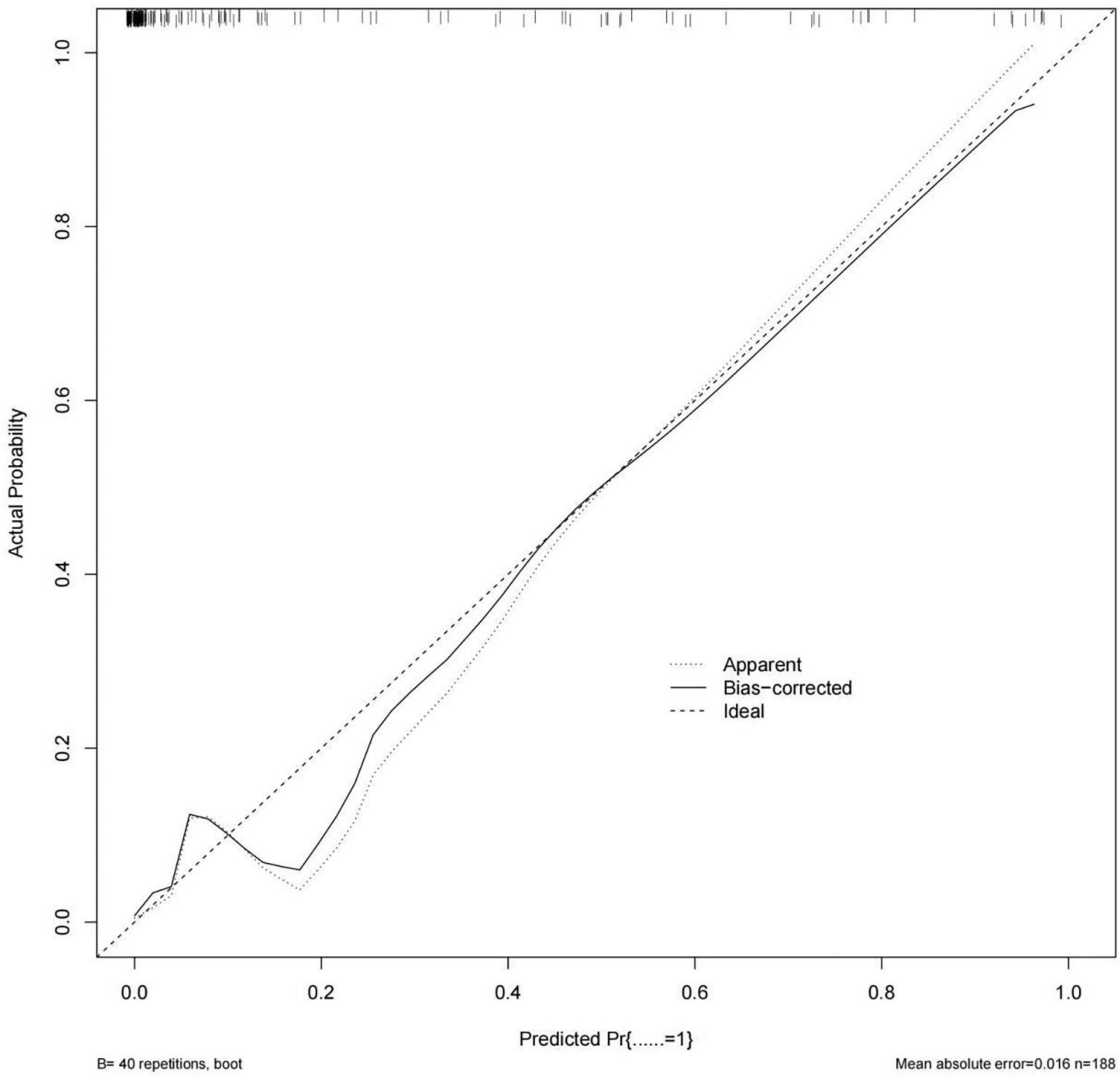
Decision curve of the nomogram model for CVC-RT in critically ill children.

**Figure 5:**
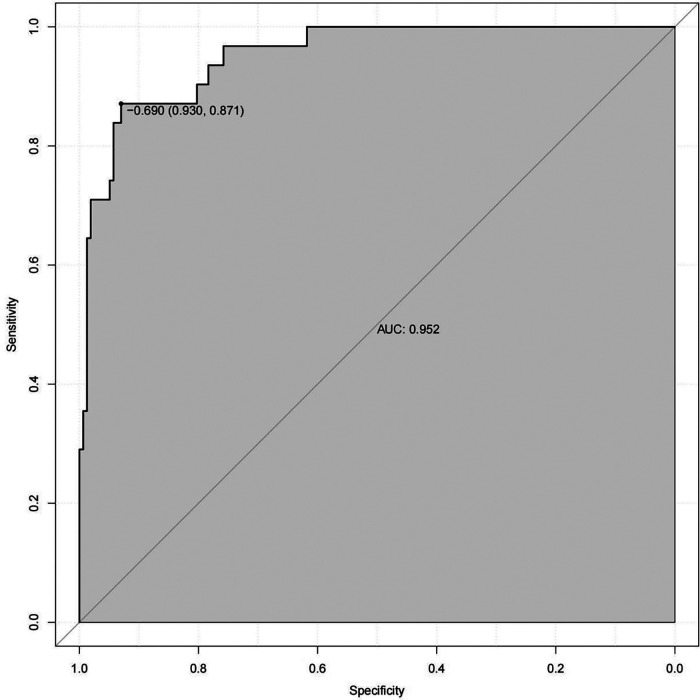
Calibration curve of the nomogram model for CVC-RT in critically ill children.

**Figure 6:**
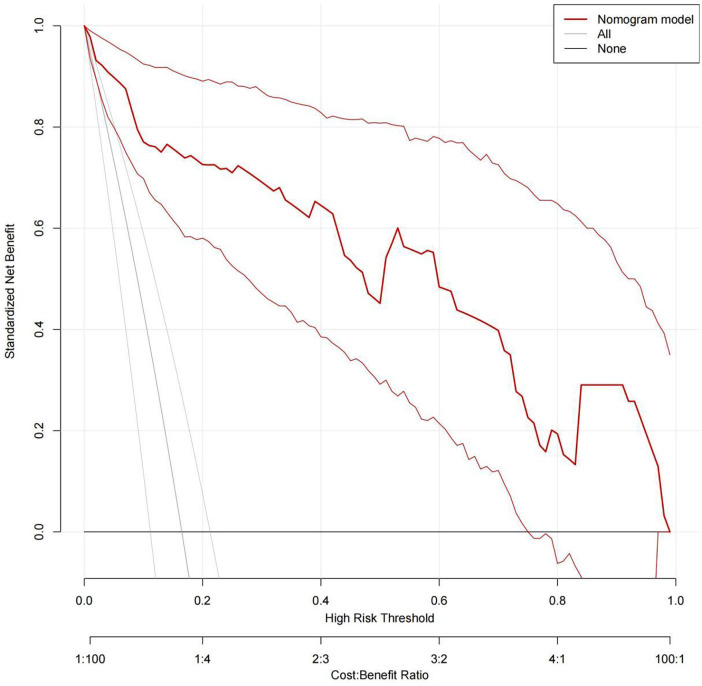
ROC curve of the nomogram model for CVC-RT in critically ill children.

Internal validation using decision curve analysis demonstrated that the model could effectively identify critically ill children at risk of CVC-RT (Figure 4). The calibration curve further indicated high agreement between predicted and observed outcomes (Figure 5). Moreover, the model exhibited excellent discriminative ability, with an area under the ROC curve of 0.952 (Figure 6).

## 4 Discussion

CVC-RT is a relatively severe complication associated with central venous catheters. Due to differences in physiology and underlying conditions between children and adults, the management of CVC-RT in critically ill pediatric patients presents greater therapeutic challenges. In this study, 31 out of 188 critically ill children developed CVC-RT, yielding an incidence rate of 16.5%. A 2011 study reported a venous thromboembolism incidence of 0.93% among 6,653 children admitted to 11 PICUs in the United States, with 88% of the VTE cases having received central venous catheters (18). This rate is substantially lower than our observed incidence. However, more recent studies have reported higher CVC-RT rates. For instance, a study by Li et al. (14) involving 1,830 critically ill children in PICUs with CVCs placed for more than three days reported a CVC-RT incidence as high as 15%. Azzam et al. (19) demonstrated a CVC-RT incidence of 5.4% among 255 PICU patients enrolled over a 17-month period. Compared with these findings, the incidence in our cohort was higher, which may be attributed to differences in screening and diagnostic strategies. Earlier studies primarily relied on ultrasound examinations performed only after clinical suspicion of thrombosis, thereby capturing mostly symptomatic events and potentially underestimating the true disease burden. In contrast, our study implemented timely ultrasound screening at an earlier stage, enabling the detection of more subclinical or asymptomatic thrombotic events. However, as highlighted by Jones et al. (20), many asymptomatic central venous catheter-related thrombi lack clinical relevance and may resolve spontaneously without intervention. Therefore, while our approach provides a more comprehensive assessment of CVC-RT occurrence, it is critical to distinguish between clinically significant and incidental findings. Our observed incidence likely reflects both clinically relevant thrombosis and transient, non-progressive events. Future studies should focus on differentiating these subtypes and evaluating their long-term clinical implications.

Additionally, this study demonstrated that patient age, catheter type, use of parenteral nutrition, D-dimer, and FIB levels were all risk factors for CVC-RT in critically ill children. Similar to our findings, Wang (21) identified age (OR = 0.755), D-dimer (OR = 9.490), and FIB as risk factors for CVC-RT occurrence in critically ill children. In terms of age, our study found that the age of children with CVC-RT was significantly lower than those without CVC-RT. A cohort study of Ketan et al. (22) found that younger age was one of the independent risk factors associated with an increased risk of CVC complications in children. Wang (21) similarly found that the age of children with CVC-RT was lower than that of children without CVC-RT. We speculated that the blood vessels of younger children are more slender; after CVC insertion, the mechanical compression on the vascular wall is more pronounced, which easily causes endothelial injury, and increases the risk of thrombosis. However, a study on the incidence of catheter-related venous thrombosis in non-critically ill children found that an increase in age was associated with an increased risk of thrombosis, which is contrary to our findings (23).

Parenteral nutrition and catheter type were also identified as risk factors for CVC-RT in the primary analysis of our total cohort. The association between parenteral nutrition and an elevated risk of CVC-RT in children is well-established (24). Furthermore, children dependent on parenteral nutrition appear more susceptible to recurrent CVC-RT (25).Consistent with our initial findings, a meta-analysis by Fu et al. (26) confirmed catheter type as a risk factor for CVC-RT in hospitalized children, although that analysis did not evaluate the risk associated with specific catheter types. In our study, the catheter types used in the thrombosis group were predominantly large-bore catheters (especially 6.6F), which we hypothesize was a key contributor to the observed higher CVC-RT incidence. The thrombosis cohort had a mean age of 0.82 ± 0.29 years. Given the extremely narrow and fragile veins in such young infants, large-bore catheters can lead to unfavorable catheter-to-vessel ratios, promoting endothelial injury and blood stasis. This mechanism likely explains the notably higher thrombosis rate in the 6.6F subgroup. However, to address concerns regarding the disproportionate influence of this high-risk subgroup, we performed a sensitivity analysis excluding all patients with 6.6F catheters. In this restricted cohort, catheter type was no longer a significant factor, and age emerged as the only independent risk factor for CVC-RT in the multivariate model. This contrast underscores that the strong association between catheter type (specifically large-bore catheters) and thrombosis in the full cohort was heavily driven by the high-risk profile of young infants receiving 6.6F catheters for critical indications. It highlights that catheter size, particularly in the context of small infant vasculature, is a critical modifier of thrombosis risk. Therefore, while our overall findings align with previous reports on catheter type as a risk factor, the sensitivity analysis clarifies that this risk is not uniform and is substantially concentrated in specific, high-risk scenarios involving large catheters in very young children.

In this present study, we also observed that both pre-insertion D-dimer and FIB levels were independently associated with CVC-RT occurrence in critically ill children, with higher levels noted in the thrombosis group compared to the non-thrombosis group. This association suggests that elevated pre-procedural D-dimer and FIB may reflect a pre-existing hypercoagulable state, which could contribute to thrombus formation following catheter placement. Consistent with previous reports, elevated D-dimer and FIB have been identified as risk markers for CVC-RT in pediatric populations (12, 19, 26). However, it is important to note that our study only measured D-dimer levels prior to CVC insertion, without serial daily measurements thereafter. Therefore, while these biomarkers are associated with increased thrombosis risk, we cannot infer a direct causal relationship or determine whether their elevation preceded or followed thrombus initiation. The median time to thrombosis diagnosis was 7 days, and in the absence of longitudinal biomarker data, the temporal dynamics between D-dimer elevation and thrombus formation remain unclear. Thus, our findings support the utility of pre-insertion D-dimer and FIB as predictive markers for identifying children at higher risk of CVC-RT, rather than establishing them as causative factors. Future studies incorporating serial biomarker measurements are needed to clarify their kinetic profiles and precise role in thrombosis pathogenesis.

Several studies have identified prolonged catheterization time as a risk factor for thrombosis (13, 26). However, our results indicated no association between catheterization time and the occurrence of CVC-RT; notably, the documented catheterization time in the thrombosis group was shorter than that in the non-thrombosis group. Kaplan–Meier analysis of thrombosis-free survival further supported this finding: stratification by catheter diameter (≤2.6F vs. 6.6F) showed a steeper decline in the 6.6F group but no statistically significant difference (log-rank *p* = 0.4422), while parenteral nutrition was associated with a significantly higher thrombosis risk (log-rank *p* = 0.0273). The core reason for this numerical discrepancy is that catheters were immediately removed or replaced upon confirmation of CVC-RT, resulting in truncated documented dwell time in the thrombosis group, representing a direct methodological explanation. Additionally, the risk of catheter-related thrombosis does not increase linearly with dwell time but persists throughout the typical catheter usage cycle (approximately 2 weeks), which may mitigate the expected positive association between prolonged placement and thrombosis as held by the traditional view. Furthermore, this finding aligns with the results of our multivariate logistic regression analysis, indicating that thrombus formation is more predominantly driven by intrinsic risk factors such as the patient's underlying hypercoagulable state (e.g., systemic infection, elevated inflammatory markers) and receipt of parenteral nutrition, rather than solely by the physical duration of catheter indwelling. Collectively, these factors may explain the discrepancy between our conclusions and previous reports.

Moreover, we developed a nomogram prediction model based on risk factors for CVC-RT in critically ill children, including age, catheter type, parenteral nutrition status, D-dimer, and FIB levels. The ROC curve showed an AUC of 1, indicating excellent discriminative ability of the model, and decision curve analysis confirmed its clinical utility. This model addresses the gap in pediatric CVC-RT prediction tools, and its visual design enables rapid bedside risk assessment, meeting the time-sensitive demands of critically ill children's care.

However, this study has several limitations that warrant acknowledgment. As a single-center retrospective analysis with a relatively small sample size (particularly 31 cases in the thrombosis group), our findings may have limited statistical power and generalizability. Notably, the observed 16.5% thrombosis incidence is notably higher than most published pediatric data—while potentially related to proactive asymptomatic screening, this raises concerns about external validity. Furthermore, the 6.6F catheter used in this study is unsuitable for young children (<1 year old), rarely used clinically and selected only for unavoidable indications rather than routine use. Thus, findings specific to this subgroup may have limited generalizability, given the link between catheter size and thrombosis risk and inter-institutional selection variations in high-risk pediatric populations. Additionally, the predominant use of large-diameter catheters in young infants (mean age 0.82 ± 0.29 years) likely contributed to elevated risk, as the suboptimal catheter-to-vessel ratio may induce endothelial injury in their narrow, fragile veins. Consistent with institutional protocol, routine prophylactic anticoagulation was not administered, which may have influenced thrombosis rates and restricts the model's applicability to settings with standard anticoagulant use. Undiagnosed hypercoagulable conditions could also confound results despite excluding children with known prethrombotic states. Furthermore, key clinical variables (e.g., PICU vs. cardiac ICU cohort, oncologic/surgical status, vasoactive scores, mechanical ventilation, lactate levels) were not routinely collected, limiting the comprehensiveness of risk factor analysis. Finally, non-standardized timing and frequency of ultrasound screening may have introduced variability in asymptomatic thrombosis detection.

Future studies should aim to validate our nomogram model in larger, multi-center prospective cohorts to enhance its generalizability and clinical applicability. Incorporating anticoagulation use as a key variable will be essential to clarify its role in mitigating CVC-RT risk and to refine the predictive model accordingly. Additionally, exploring the impact of specific catheter materials, insertion techniques, and the role of dynamic coagulation monitoring such as thromboelastography may further improve risk stratification. Long-term follow-up studies are also needed to assess the clinical outcomes of children identified as high-risk by the model, including the efficacy of early intervention strategies.

In conclusion, our study demonstrated that age, catheter type, parenteral nutrition, D-dimer, and FIB levels were risk factors for CVC-RT in critically ill children, and the nomogram prediction model constructed based on these factors exhibited good discriminative ability and high predictive value. This study provides actionable insights for clinical guidance, though formal practice changes at our institution await prospective validation. The nomogram presents a tool for potential bedside risk stratification, while the link between pre-insertion D-dimer/FIB levels and CVC-RT risk supports their utility in identifying high-risk patients. Our findings also reinforce the importance of selecting the smallest feasible catheter, especially in young infants. Future work will focus on validating the nomogram in larger cohorts and assessing the impact of these risk-stratified approaches on clinical outcomes.

## Data Availability

The raw data supporting the conclusions of this article will be made available by the authors, without undue reservation.
