# Supplementary material for: Development of a risk prediction model for central venous catheter insertion-related thrombosis in critically ill pediatric patients
Source: Front Pediatr. 2026 Mar 24;14:1666896. doi: 10.3389/fped.2026.1666896 (PMC13054883; doi:10.3389/fped.2026.1666896)
Supplement: Supplementary file 1 [file Table1.docx]

Table 1 Comparison of baseline characteristics in critically ill children [n (%)] (x̄ ± s)

| Project | Non-thrombosis group (n=157) | Thrombosis group  (n=31) | Z/χ2/t | P value |
| --- | --- | --- | --- | --- |
| age (years) | 4.96±0.52 | 0.82±0.29 | 42.92 | <0.0001 |
| gender |  |  | 0.1317 | 0.895 |
| male | 78（49.7%） | 15（48.4%） |  |  |
| female | 79（50.3%） | 16（51.6%） |  |  |
| disease type |  |  | 3.880 | 0.5668 |
| nervous system | 58（36.9%） | 11（35.5%） |  |  |
| respiratory system | 34（21.7%） | 6（19.4%） |  |  |
| sepsis | 23（14.6%） | 7（22.6%） |  |  |
| cardiovascular system | 18（11.5%） | 2（6.5%） |  |  |
| tumor | 4（2.5%） | 2（6.5%） |  |  |
| others | 20（12.7%） | 2（6.5%） |  |  |
