# Supplementary material for: Development of a risk prediction model for central venous catheter insertion-related thrombosis in critically ill pediatric patients
Source: Front Pediatr. 2026 Mar 24;14:1666896. doi: 10.3389/fped.2026.1666896 (PMC13054883; doi:10.3389/fped.2026.1666896)
Supplement: Supplementary file 2 [file Table2.docx]

Table 2 Univariate analysis of CVC-RT occurrence in critically ill children [n (%)] (x±s)

| Project | Non-thrombosis group (n=157) | Thrombosis group  (n=31) | Z/χ2/t | P value |
| --- | --- | --- | --- | --- |
| catheterized side |  |  | 0.5303 | 0.5959 |
| left | 48（30.6%） | 8（25.8%） |  |  |
| right | 109（69.4%） | 23（74.2%） |  |  |
| catheter type |  |  | 6.648 | 0.0360 |
| 1.9F | 78（49.7%） | 18（58.1%） |  |  |
| 2.6F | 63（40.1%） | 6（19.4%） |  |  |
| 6.6F | 16（10.2%） | 7（22.5%） |  |  |
| catheterization time (days) | 14.25±3.31 | 13.93±2.92 | 0.5022 | 0.6161 |
| catheter lumen |  |  | 1.924 | 0.0544 |
| single-lumen | 16（10.2%） | 7（22.6%） |  |  |
| dual-lumen | 141（89.8%） | 24（77.4%） |  |  |
| parenteral nutrition |  |  | 2.114 | 0.0345 |
| yes | 18（11.5%） | 8（25.8%） |  |  |
| no | 139（88.5%） | 23（74.2%） |  |  |
| catheter-related infection |  |  | 0.8779 | 0.3800 |
| yes | 5（3.2%） | 2（6.5%） |  |  |
| no | 152（96.8%） | 29（93.5%） |  |  |
| catheter insertion site |  |  | 1.325 | 0.5155 |
| internal jugular vein | 65（41.4%） | 16（16.2%） |  |  |
| subclavian vein | 55（35.0%） | 10（6.4%） |  |  |
| femoral vein | 37（23.6%） | 5（3.2%） |  |  |
| D-dimer(mg/L) | 0.46±0.12 | 0.59±0.13 | 5.371 | <0.0001 |
| FIB(g/L) | 3.97±1.14 | 4.80±1.22 | 3.670 | 0.0003 |

Note: catheterization time refers to the total dwell time of the catheter from insertion to removal
