# Supplementary material for: Development of a risk prediction model for central venous catheter insertion-related thrombosis in critically ill pediatric patients
Source: Front Pediatr. 2026 Mar 24;14:1666896. doi: 10.3389/fped.2026.1666896 (PMC13054883; doi:10.3389/fped.2026.1666896)
Supplement: Supplementary file 3 [file Table3.docx]

Table 3 Multivariate logistic regression analysis of CVC-RT occurrence in critically ill children

| Independent variable | β | SE | OR | Wald χ2 | 95%CI | P |
| --- | --- | --- | --- | --- | --- | --- |
| Age | -1.543 | 0.355 | 0.214 | 18.919 | 0.107-0.428 | 0.0001 |
| Catheter type | -0.464 | 0.387 | 0.629 | 1.438 | 0.295-1.342 | 0.230 |
| Parenteral nutrition | 2.344 | 0.859 | 10.424 | 7.441 | 1.934-56.175 | 0.006 |
| D-dimer(mg/L) | 11.804 | 3.148 | 1.34e+5 | 14.064 | 280.1-6.40e+7 | 0.0001 |
| FIB(g/L) | 0.545 | 0.262 | 1.724 | 4.330 | 1.032-2.880 | 0.037 |
