# Supplementary material for: Development of a risk prediction model for central venous catheter insertion-related thrombosis in critically ill pediatric patients
Source: Front Pediatr. 2026 Mar 24;14:1666896. doi: 10.3389/fped.2026.1666896 (PMC13054883; doi:10.3389/fped.2026.1666896)
Supplement: Supplementary file 4 [file Table4.docx]

Table S1 Subgroup analysis of factors associated with CVC-RT in critically ill children receiving 6.6F catheters

| Project | Non-thrombosis group (n=16) | Thrombosis group (n=7) | Z/χ2/t | P value |
| --- | --- | --- | --- | --- |
| age | 4.99±0.55 | 0.63±0.23 | 20.01 | <0.0001 |
| gender |  |  | 0.3155 | 0.7525 |
| male | 8 | 3 |  |  |
| female | 8 | 4 |  |  |
| catheterized side |  |  | 0.1796 | 0.8576 |
| left | 4 | 2 |  |  |
| right | 12 | 5 |  |  |
| Incidence of DVT | 7/23(30.4%) | |  |  |
