# Supplementary material for: Development of a risk prediction model for central venous catheter insertion-related thrombosis in critically ill pediatric patients
Source: Front Pediatr. 2026 Mar 24;14:1666896. doi: 10.3389/fped.2026.1666896 (PMC13054883; doi:10.3389/fped.2026.1666896)
Supplement: Supplementary file 5 [file Table5.docx]

Table S2 Assignment method of independent variables

| Independent variable | Assignment method |
| --- | --- |
| Age | Continuous variables were entered as actual values |
| Catheter type | Dummy variables were assigned to the categorical variable as follows: 1.9F = 1, 2.6F = 2, 6.6F = 3. |
| Parenteral nutrition | Yes=1, No=0 |
| D-dimer(mg/L) | Continuous variables were entered as actual values |
| FIB(g/L) | Continuous variables were entered as actual values |
